# Cost-effectiveness of cardiovascular risk management by practice nurses in primary care

**DOI:** 10.1186/1471-2458-13-148

**Published:** 2013-02-18

**Authors:** Ans H Tiessen, Karin M Vermeulen, Jan Broer, Andries J Smit, Klaas van der Meer

**Affiliations:** 1 University Medical Center Groningen, Department General Practice, University of Groningen, Groningen, The Netherlands; 2 University Medical Center Groningen, Department Epidemiology, University of Groningen, Groningen, The Netherlands; 3 Municipal Public Health Service Groningen, Groningen, The Netherlands; 4 University Medical Center Groningen, Department Internal Medicine, University of Groningen, Groningen, The Netherlands

**Keywords:** Cost-effectiveness, Arteriosclerosis, Cardiovascular diseases, Primary health care, Prevention and control, Self-management

## Abstract

**Background:**

Cardiovascular disease (CVD) is largely preventable and prevention expenditures are relatively low. The randomised controlled SPRING-trial (SPRING-RCT) shows that cardiovascular risk management by practice nurses in general practice with and without self-monitoring both decreases cardiovascular risk, with no additional effect of self-monitoring. For considering future approaches of cardiovascular risk reduction, cost effectiveness analyses of regular care and additional self-monitoring are performed from a societal perspective on data from the SPRING-RCT.

**Methods:**

Direct medical and productivity costs are analysed alongside the SPRING-RCT, studying 179 participants (men aged 50–75 years, women aged 55–75 years), with an elevated cardiovascular risk, in 20 general practices in the Netherlands. Standard cardiovascular treatment according to Dutch guidelines is compared with additional counselling based on self-monitoring at home (pedometer, weighing scale and/ or blood pressure device) both by trained practice nurses. Cost-effectiveness is evaluated for both treatment groups and patient categories (age, sex, education).

**Results:**

Costs are €98 and €187 per percentage decrease in 10-year cardiovascular mortality estimation, for the control and intervention group respectively. In both groups lost productivity causes the majority of the costs. The incremental cost-effectiveness ratio is approximately €1100 (95% CI: -5157 to 6150). Self-monitoring may be cost effective for females and higher educated participants, however confidence intervals are wide.

**Conclusions:**

In this study population, regular treatment is more cost effective than counselling based on self-monitoring, with the majority of costs caused by lost productivity.

**Trial registration:**

Trialregister.nl identifier: http://NTR2188

## Background

Cardiovascular diseases (CVDs) are a leading cause of death both worldwide (29% of all deaths, 2004) [1] as in the Netherlands (28% of all deaths, 2011; Statistics Netherlands). CVDs are to a large extent preventable [2]. In 2009, in Dutch family practice, €39 million was spent on non-pharmaceutical cardiovascular preventive activities, i.e. risk profiling, blood pressure (BP) measurements and lifestyle counselling [3]. Annual costs at patient level were estimated for the Dutch situation, including doctor’s visits, repeat prescriptions, drug costs and excluding diagnostics and productivity costs, as €293 for statin use and €258 for antihypertensives [4].

Lifestyle interventions appear to be cost effective in reducing cardiovascular risk [5]. A Dutch study found that preventive cardiac medication, following a 2006 revised guideline, was also cost effective [4]. This guideline recommended a broader indication for starting medication than previous guidelines and was also the basis for SPRING study (Self-monitoring and Prevention of RIsk factors by Nurse practitioners in the region of Groningen), of which the cost effectiveness is discussed in this paper. Drug treatment of hypertension and dyslipidaemia for cardiovascular prevention according to Canadian guidelines appeared to be cost effective as well [6]. However, Grover et al. concluded that treatment is inefficient for younger individuals [6]. Kok et al. concluded that -although effects are more pronounced in older age groups- health gain per prevented case is larger for all age groups up to the age of 70 [4]. Several studies on cost-effectiveness of combined screening, lifestyle and pharmaceutical interventions in (cooperation with) primary care indicated increased life expectancy without increased costs [7-10], were inconclusive in one study, [11] or were depending on differences in assumptions on long-term duration of effect [12-14]. Most of the previously mentioned studies did not use a societal perspective (i.e. taking into account absence at work and transportation of participants), except Rasmussen and Salkeld [7-14]. Rasmussen and Salkeld took into account prevented productivity losses for working individuals experiencing a cardiovascular event, but did not take into account productivity losses due to the intervention [7,11]. So, there is a need for cost effectiveness-analysis of cardiovascular preventive interventions in general practice from a societal point of view.

For the SPRING-RCT we reported that combined lifestyle and drug intervention by practice nurses in persons with a mild to moderate cardiovascular risk resulted in a small but significant decrease of cardiovascular risk after one year (∆SCORE 10 year risk of cardiovascular mortality –1.71% (SD 2.95) for both groups together), [15] without significant additional effect of intensive treatment with self-monitoring compared with standard treatment [16]. Interventions that cause modest risk reduction in individuals, like the SPRING trial, may have a substantial cumulative effect when applied on the population as a whole (prevention paradox). Before implementation of such an intervention program, costs and cost-effectiveness should be considered.

The objectives of this paper are to assess the costs and cost-effectiveness of cardiovascular prevention by practice nurses from a societal perspective in both treatment groups of the SPRING-RCT, and specify the costs of components of the intervention and the cost-effectiveness for subgroups (age, sex, level of education).

## Method

### SPRING population and intervention

The economic evaluation was conducted using data gathered in the SPRING-RCT. Between June 2008 and August 2009 randomly selected individuals from 20 general practices (GPs) were invited for a screening. Men aged 50–75 years and women aged 55–75 years, with an estimated SCORE 10-year cardiovascular risk of cardiovascular mortality ≥5% [15], at least one treatable risk factor (smoking, hypertension, lack of physical activity or overweight) and without history of CVD, diabetes mellitus, thyroid dysfunction or an estimated life expectancy <2 years, were randomised at patient level into two groups. The control group received standard treatment according the 2006 Dutch general practitioner’s guideline, [17] conducted by specially trained practice nurses. The intervention group additionally received counselling based on self-monitoring at home, with pedometers, weighing scales and/or BP devices. After one year 179 participants were analysed, the primary outcome was the SCORE cardiovascular risk estimation. The mean effect for the control group was 1,63% decrease in SCORE 10-year risk of fatal CVD; the mean decrease for the intervention group was 1,79%. The SPRING study was approved by the Medical Ethics Review Committee of the University Medical Centre Groningen (reference number 2007/232). For more information on study design and outcomes, see Tiessen et al [16].

### Cost calculation

Total costs were calculated for both treatment groups. Total costs consisted of direct costs (medication, time spent by medical staff, self-monitoring equipment, patient transport to the practice) and productivity losses (absence at work of the working individuals that participated). Costs of intangibles (suffering/adverse effects) and medical costs not directly related to the study were not taken into account.

The staff costs of the visits to the practice nurse were based on the registered total visit time and standard hourly costs of practice nurses in general practice in the Netherlands (Table 1). As the practice nurses reported that time spent on consultation with the general practitioner about participants was negligible, this was not taken into account. Productivity costs of participants were calculated for participants that were employed in a paid job and were based on duration of the visits plus 30 minutes/visit as transportation time, using mean salary costs stratified by gender [18]. Transportation costs to the practice for the participants were based on the average distance for Dutch inhabitants to their GP’s of 1.1 km [18]. Prices of pedometers, weighing scales and BP devices were based on average customer prices for these devices and were calculated for participants who had actually used the self-monitoring devices. Only medication that was newly added or for which the dosage was adapted during the treatment, was taken into account. 2012 prices of generic medication were used, including value added tax, and a pharmacists allowance for every 3 months.

**Table 1:** Economic variables

	**€**	**Reference/data source**
Visit practice nurse (€/minute)	0.85	Vektis, information centre on costs and quality of health care in the Netherlands, established by health care insurers.
Mean salary costs men (€/hour)	32.49	Manual Hakkaart-van Roijen et al. [18]
Mean salary costs women (€/hour)	25.94	Manual Hakkaart-van Roijen et al. [18]
Transportation costs per visit	3.44	1.1 km * €0.20/km (back and forth: €0.44) + €3 parking costs, based on the manual of Hakkaart-van Roijen et al. [18]
Weighing scale	45	Mean price according to the “Consumentenbond” (Dutch consumers organisation)
Pedometer	40	Mean price according to “thuisvergelijken.nl” (website with reviews on web shops)
Blood pressure device	120	Mean price according to “bloeddrukmetershop.nl”
Food diary and step diary	5	Price according to “weightwatchers.nl”
Medication	variable	2012 prices according to “medicijnkosten.nl”, site of the College of Health Insurances

### Cost-effectiveness analysis

In order to get an impression of the balance between additional costs that have to be invested with the new risk management strategy, and the health gains of this strategy, a cost effectiveness analysis was performed. The effectiveness measure was the SCORE cardiovascular mortality estimation (expressed as % 10-year cardiovascular mortality risk). As the duration of the study was one year, costs were not discounted (discounting is common in cost effectiveness studies with a longer duration, when costs are made at the start and future benefits are expressed in today’s values). Cost effectiveness ratios (CERs) in €/% SCORE were calculated for both treatment groups separately. Incremental cost effectiveness ratios (ICERs) (expressed as difference in costs between the two methods/ difference in % SCORE) were calculated to compare the standard treatment and the intervention treatment from the SPRING-RCT with regard to both costs and effects. In addition, subgroup analyses were performed for different patient categories. For both groups cost and effect pairs were bootstrapped 5000 times from the trial data, which means that based on a ‘random sample with replacement’ from the original trial data, 5000 new data sets were drawn, that were used to estimate the ICER 95% confidence interval. In addition, a cost effectiveness plane was constructed to graphically display the location of the estimates (right upper quadrant: more effective and more expensive, right lower quadrant: more effective and less expensive, left upper quadrant: less effective and more expensive and left lower quadrant: less effective and less expensive) [19]. Finally a cost effectiveness-acceptability curve was computed to estimate the change the new risk management strategy is cost effective given an rage of different amounts of money a decision maker might be willing to pay for one additional unit of health [20].

## Results

Table 2 shows average costs per participant in both study groups and the contribution of different treatment aspects on total costs. Based on the mean effect of 1,63% and 1,79% decrease in SCORE risk estimation for the control and intervention group respectively, [16] the CERs are €98 and €187/% decrease SCORE risk estimation.

**Table 2:** Total costs and costs of different aspects of the treatment for complete cases in both groups per year (€)

	**Control group**	**Intervention group**
	**(N = 89)**	**(N = 87)**
**Total cost per individual**	160	335
**Productivity of participants**	72	139
**Transport to practice for participants**	9	17
**Medical staff**	55	110
**Self-monitoring equipment**	10	44
**Medication adjustments**	15	25
**Statin**	3	11
**Thiazides**	11	10
**Beta blocker**	0	1
**Calcium antagonist**	0	1
ACE-inhibitor*/Angiotensin II antagonist	0	2

*ACE = angiotensin converting enzyme.

**Figure 1:**
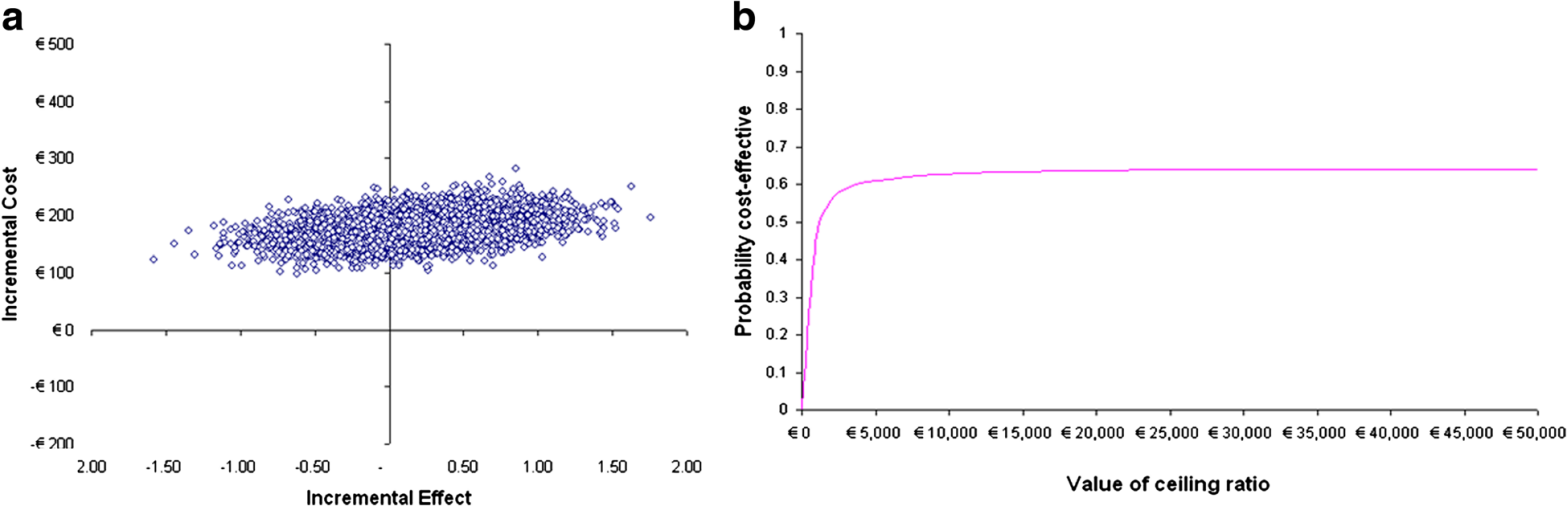
Scatter plot of bootstrapped costs and effects (a) and value of ceiling ratio (b) for the whole study population.

The mean difference between both groups regarding treatment effect is 0.16% (95% CI −1.66 – 1.98) SCORE risk estimation. The mean difference regarding costs is €175 (95% CI 80–270). The ICER, therefore, is €1082, which means that for the intervention group approximately €1100 has to be invested extra to obtain 1% extra decrease of SCORE risk estimation. The 95% confidence interval for the ICER based on 5000 bootstrap replications is -€5157 to €6150. The replications are depicted as a cost effectiveness plane (Figure 1a). The location of the incremental cost effectiveness pairs is above the horizontal axis, which indicates the higher costs with the intervention group treatment compared with the control group treatment. With regard to the effectiveness, there is considerable uncertainty: 35% of the pairs are left from the y-axis, indicating more effect for the control group treatment and 65% are right from the y-axis, indicating more effect of the intervention group treatment. Figure 1b depicts that irrespective of the amount a decision maker is willing to pay, the probability of the intervention treatment being more cost effective than the control treatment is at most 60%.

**Figure 2:**
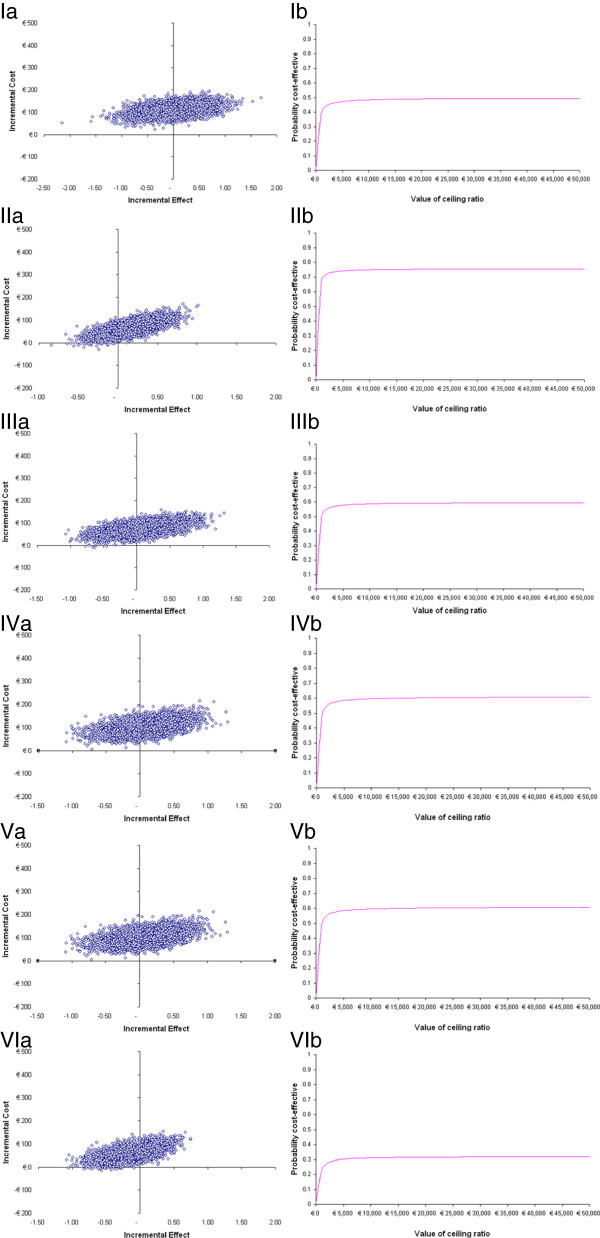
Scatter plot of bootstrapped costs and effects (a) and value of ceiling ratio (b) for subgroups: I: males, II: females, III: age ≤65y, IV: age >65y, V:low educated individuals, VI: high educated individuals.

Table 3 shows CER’s and ICER’s for subgroups. For all subgroups the cost effectiveness ratio (CER) is lower for the control treatment compared with the intervention treatment. The two columns at the right side of the table illustrate the relative cost and effect of the intervention group compared with the control group for the subgroups. ICERs for the subgroup pairs show that the intervention treatment was most cost effective for females and participants aged >65 years and to a lesser extent also for higher educated participants. However, confidence intervals are very large. The results for the subgroups are also depicted as cost effectiveness planes and value of ceiling ratios in Figure 2.

**Table 3:** Effect (Δ% SCORE* risk assessment), cost (€), CER* (€/% SCORE) and ICER* (Δ €/ Δ**% SCORE) in specific subgroups**

		**Control group**			**Intervention group**			**ICER (95% CI)**	**Effect**	**Costs**
		**Effect**	**Costs**	**CER**	**Effect**	**Costs**	**CER**			
**Sex**	♂	1,7	155	91	1,6	301	187	−1578 (−39811 – 41036)	-	+
	♀	1,5	171	114	2,3	420	185	325 (−2662 – 2601)	++	+
**Age**	**≤65y**	1,8	184	102	1,8	303	167	5865 (−3257 – 3363)	+	+
	**>65y**	1,5	133	92	1,8	376	213	768 (−4076 – 4759)	+	+
Educ.*	**low**	2,4	174	74	1,8	369	208	−340 (−3649 – 3793)	--	+
	**high**	1,3	157	121	1,8	319	182	351 (−3402 – 2923)	+	+

*SCORE = systematic coronary risk evaluation (estimated 10-year cardiovascular mortality risk), CER = cost effectiveness ratio, ICER = incremental cost effectiveness ratio, Educ. = educational level.

## Discussion

### Main findings

In this population, the costs of cardiovascular prevention were higher in the intervention group, with annual costs per individual of €160 (control group) compared with €335 (intervention group). Costs per percent decrease in estimated 10-year cardiovascular mortality of €98 compared with €187, for the control and intervention group respectively. An added role for self-monitoring can be considered only for females and higher educated individuals. For both groups costs predominantly consisted of societal costs and staff time and not of medication.

### Explanation and comparison with existing literature

The present study adds valuable new information compared with previous studies, as both costs and effects are based on an actual practice setting, which makes the outcomes more generalizable. In addition, societal costs were included, which is recommended to allow a broad perspective [21]. Lost productivity due to practice visits caused the majority of costs and is usually not taken into account.

The time investment of both medical staff and participants not only caused the main cost driver in both groups, but also the difference between both groups (Table 1). The number of visits of intervention group participants was almost twice the number of the control group participants and also the duration per visit was slightly longer in the intervention group [16]. All intervention group participants were offered lifestyle counselling and home collected self-monitoring results were discussed during the visits. The productivity cost estimates may have been on the high side with 30 minutes transportation time, and with some working individuals probably having part-time jobs with planned visits during spare time. Of minor influence were the increased costs of self-monitoring equipment and medication adjustments in the intervention group. Despite self-monitoring not being part of the control group treatment, some self-monitoring costs were made due to BP measurements (on participant’s initiative) at home.

Based on De Bekker-Grob et al. with €39 million spend on non-pharmaceutical cardiovascular preventive activities and €181 million on medication, we expected that costs of modified medication would be relatively high [3]. In the present study however, these had only minor influence and consisted in both groups mainly of statins and thiazides. Medication costs in the SPRING study may be even overestimated, as we calculated costs as if all adjusted medication was prescribed for the whole study period whereas mostly participants were advised first to adjust their lifestyles. De Bekker-Grob et al. found a large difference in medication prescription between different GP’s. We did not study the differences in prescriptions between practices, but this probably is of minor influence in this study because medication adjustments were advised by the study protocol. As mentioned before, Kok et al. estimated for the Dutch situation annual costs as €293 for statin use and €258 for antihypertensives [4]. Compared with the Kok et al. the costs in the SPRING-RCT appear to be much lower, despite in the SPRING-RCT societal costs were taken into account. The goal of Kok et al. was not to estimate annual costs however, it was one step in estimating the cost-effectiveness of a new guideline. It is hard to make exact comparisons with other studies due to differences in programs, perspective and whether statins had already run out of patent (which reduces costs significantly) or not.

With regard to exploration of the subgroups, the control group CER is lower for all subgroups. Higher educated participants and women seem to benefit most from the investment of extra time and immediate feedback and motivation from self-monitoring, as the ICER is most favourable for these two groups. Whether the intervention programme is preferable over control treatment for these groups, depends on how much a decision maker is willing to pay for a certain decrease in SCORE risk estimation. However, confidence intervals were very wide and not statistically significant.

On the other hand, especially lower educated participants seemed to be better off in the control group. A probable explanation is that for some lower educated individuals the instructions and feedback of the self-monitoring might have been too complex and might have had a discouraging effect. Higher social economic status is inversely related to cardiovascular risk [22-27]. Some investigators suggest that screening and treating high risk individual patients might augment socio-economic health differences, compared with whole-population approaches [28]. Self-monitoring probably enhances these differences. Individuals from a higher socio-economic background who are motivated for using self-monitoring might be asked to pay a contribution. During the SPRING-RCT, participants were offered self-monitoring free of charge, but self-monitoring devices are usually not reimbursed by health insurance companies in the Netherlands.

With respect to sex, the awareness of both the public and physicians is poor about the fact that -despite women having lower 10-year risk estimations compared with men- the annual death rate for CVDs is higher among females compared with males, due to a higher case fatality rate from coronary attacks [1,29]. Reduction of risk factors is also effective for cardiovascular risk in women [30]. Our study indicates that self-monitoring may improve cardiovascular risk management in women.

There is controversy about cardiovascular risk management for specific age groups [4,6]. Both these studies estimated long term effects and our study only evaluates effects after one year. For this study, the intervention group CER is higher for participants aged 65 years and older, compared with younger participants, despite the fact that productivity losses will be present mostly in younger participants. Incremental cost-effectiveness ratio is more favourable for participants aged 65 years and older.

### Strengths and limitations

Strengths of this study are the pragmatic protocol and the societal perspective. No information from the SPRING study was available on long term effects >1 year nor on adverse effects. No modelling was performed to estimate for example quality adjusted life years, which makes comparisons with some other studies difficult. On the other hand the lack of assumptions necessary for modelling makes the results more plain to interpret. The group size was too limited to allow reliable cost effectiveness analyses in subgroups, so conclusions about subgroups should be considered as preliminary.

## Conclusion

In the studied population, standard cardiovascular risk management by practice nurses is more cost effective than additional intensive counselling based on self-monitoring. As a considerable proportion of the target population for cardiovascular risk management will consist of working individuals, productivity losses during practice visits have to be taken into account when deciding about cardiovascular risk management strategies. The majority of costs resulted from societal costs. The costs for cardiovascular risk management however are found to be relatively low, for protocols based on the Dutch General Practitioner’s Guideline on Cardiovascular Risk Management (version 2006) [17]. Our findings support the use of this guideline for the targeted individuals in general practice. Costs from the perspective of the patient or the general practices are even considerably lower. A self-monitoring approach may be of interest for subgroups like females, facing a higher case fatality ratio. However, effects on subgroups should be further investigated, together with investigations on long term effects.

### Ethics approval

The SPRING study was approved by the Medical Ethics Review Committee of the University Medical Centre Groningen: 2007/232.

## Abbreviations

SCORE: Systematic coronary risk evaluation; SPRING: Self-monitoring and prevention of RIsk factors by nurse practitioners in the region of Groningen; RCT: Randomized controlled trial; CVD: Cardiovascular disease; BP: Blood pressure; GP: General practitioner; CER: Cost effectiveness ratio; ICER: Incremental cost effectiveness ratio

## Competing interests

No potential conflicts of interest were present.

## Authors’ contributions

AS, JB, KM and AT designed the study, AT collected the data, KV analysed the data, AT drafted the article, all other authors revised it and gave final approval of the version to be published.

## Pre-publication history

The pre-publication history for this paper can be accessed here:

http://www.biomedcentral.com/1471-2458/13/148/prepub
